# Universal screening for drug use in urban primary care

**DOI:** 10.1186/1940-0640-7-S1-A14

**Published:** 2012-10-09

**Authors:** Richard Saitz, Daniel Alford, Julie Witas, Donald Allensworth-Davies, Tibor Palfai, Debbie Cheng, Judith Bernstein, Jeffrey Samet

**Affiliations:** 1 Boston University School of Medicine and Boston Medical Center, Boston, MA, USA

## 

We describe universal screening in a large urban hospital-based primary care practice. Trained staff aimed to screen all adult patients presenting for primary care visits between July 2010 and February 2011. Screening included three items about past three-month drug use: frequency of heavy drinking (>3 standard drinks in a day for women and >4 for men); any use of prescription sedatives, opioids, or amphetamines without a prescription or in greater amounts than prescribed; and any use of illicit drugs. A convenience sample of those who screened positive for drug use was evaluated for eligibility to be in a study of brief counseling efficacy; enrollment resulted in further research assessment. During six months, 15,818 patients arrived for primary care appointments. Of these, 5549 (35%) were screened, 539 (10%) of whom reported heavy drinking (5.9% of those screened) or drug use (6.2% of those screened). Of the 539, 41% reported drug use only, 38% heavy drinking only, and 21% reported both. Of patients meeting eligibility criteria for the brief counseling study for drug use (current drug use, not pregnant, able to provide two contacts for follow-up, willing to return for research assessments, spoke English), 14% had lower risk scores (2 or 3) on the Alcohol, Smoking, and Substance Involvement Screening Test (ASSIST). Of those with scores of 4 or more (moderate risk), marijuana was the drug of most concern for 61%, cocaine for 19%, and opioids for 18%. Twenty-one percent reported prescription drug misuse, 31% used more than one drug, 20% had high-risk ASSIST scores (27+), and 48% met criteria for drug dependence. In-person universal screening in busy urban primary care settings reaches some but misses many. Drug use prevalence was 6.2%, most of which was marijuana, and many of those screened had drug dependence. Most drug use would have been missed if drug screening had been done only for those who screened positive for heavy drinking.

